# Responses of Outdoor Housed Dairy Cows to Shade Access during the Prepartum Period under Temperate Summer Conditions

**DOI:** 10.3390/ani11102911

**Published:** 2021-10-08

**Authors:** Daniel Cartes, Ana Strappini, Fabiola Matamala, Rodrigo Held-Montaldo, Pilar Sepúlveda-Varas

**Affiliations:** 1 Escuela de Graduados, Facultad de Ciencias Veterinarias, Universidad Austral de Chile, Valdivia 5090000, Chile; dcarteslillo@gmail.com (D.C.); fabiolamatamalahernandez@gmail.com (F.M.); rodrigo.heldm@gmail.com (R.H.-M.); 2 Instituto de Ciencia Animal, Universidad Austral de Chile, Valdivia 5090000, Chile; anastrappini@uach.cl; 3 Instituto de Ciencias Clínicas Veterinarias, Universidad Austral de Chile, Valdivia 5090000, Chile

**Keywords:** animal welfare, transition period, behavior, shade

## Abstract

**Simple Summary:**

Late pregnant dairy cows housed outdoor can be exposed to hot weather conditions for several weeks prior to calving affecting their physiology and behavior. We aimed to determine whether access to an artificial shade for outdoor-housed dairy cows during the three weeks prior to calving had a positive effect on lying, rumination, feeding, and drinking behaviors. Also, the relationship between access to shade and health status was investigated. Shaded cows increased rumination time, but the daily lying time was similar to unshaded cows. Also, shaded cows spent half of the time drinking during the warmest hours of the day and spent more time feeding during the morning feed than unshaded cows. The prepartum and postpartum body fat mobilization and presentations of clinical diseases after calving were similar between both treatments. This study presents evidence that shade is an important resource for cows during temperate summers, observing effects mainly on behavioral variables.

**Abstract:**

Cows are affected by environmental factors associated with warm weather conditions; however, little is known about the effect of shade access especially during the prepartum period of dairy cows in temperate regions. This study assessed the effect of shade on the behavior (lying, rumination, feeding, and drinking), body fat mobilization, and health status of outdoor-housed dairy cows during the prepartum period under temperate summer conditions. During the 3 weeks prior to calving, 24 multiparous Holstein cows were grouped (4 cows/group) and assigned to either an open corral without shade or with access to shade until calving. We daily measured shade use, lying, rumination, feeding, and drinking behavior. Weekly, prepartum non-esterified fatty acids (NEFA) and postpartum b-hydroxybutyrate (BHB) concentrations were measured. Clinical examination was periodically performed individually until 21 postpartum days. Shade use averaged 45.6, 46.0, and 19.8% during the hottest hours of the day (11–18 h) in weeks 3, 2, and 1 prior to calving, respectively. Shaded cows had higher values for rumination time and feeding time during the morning but spent less time drinking during the warmest hours than unshaded cows. NEFA and BHB concentrations and clinical diseases were similar between both treatments. These findings suggest that under temperate summer conditions the access to an artificial shade is an important resource, observing beneficial effects mainly on behavioral variables.

## 1. Introduction

Seasonal calving is often used in grazing dairy farms in temperate regions to maximize pasture nutrient utilization to support lactation, minimize costs associated with purchased feeds and maintain the supply of milk through the year [1,2,3]. Cows that calve at the beginning of the autumn calving season might be especially affected by environmental factors associated with warm summer weather during their prepartum periods such as high air temperature, relative humidity, and high solar radiation levels [4]. Considerable research has shown that exposure to warm weather imposes challenges to the welfare and productivity of animals in pasture-based systems, but most of the work to date has focused on lactating dairy cows [5,6,7]. A better understanding of the responses of dry pregnant cows to summer weather even in temperate zones would be an important first step for designing better management practices during this important period.

Although behavioral changes have been documented in both indoor and pasture lactating dairy cows exposed to warm weather, little is known about how these climatic conditions may affect cow behavior during the weeks before calving. Karimi et al. [8] showed that prepartum housed cows adapt to heat stress (defined as the temperature–humidity index, THI > 72, equivalent to 25 °C at 50% relative humidity) through increasing meal size and reducing meal duration, as well as increasing standing times. Similarly, Paudyal et al. [9] reported that the average rumination time during the days prior to calving in indoor cows was lower in the hot season (monthly average THI ≥ 76) compared to the cool season (monthly average THI < 76). More recently, Edwards et al. [10] found that cows kept on pasture and exposed to moderate heat stress conditions (THI > 68 and ≤ 79) during the week before calving decreased their lying time compared to those not experiencing heat stress conditions (THI ≤ 68). To date, no studies have quantified the effect of warm environmental conditions on the feeding and ruminating behavior of prepartum dairy cows managed outdoors. 

Previous research on late gestating dairy cows housed indoors has also determined that hot conditions reduce their feed intake, causing body fat mobilization and weight loss [11]. Excessive lipid mobilization, reflected by an increase of non-esterified fatty acids (NEFA) and β-hydroxybutyrate (BHB) levels, contributes to oxidative stress and inflammatory responses [12,13] and increase the risk for common postpartum disorders [14,15]. Thus, exposure of prepartum cows to summer warm weather may influence their health status after calving. 

In pasture-based systems, one of the most economical ways of modifying the environment to mitigate the effect of summer weather is the provision of shade [16]. Previous studies have shown that seeking shade is an important behavioral coping strategy for pasture lactating cows when exposed to hot summer conditions in temperate climates [17,18,19,20]. For instance, shade access on sunny days with temperatures higher than 25 °C is beneficial to reduce the body temperature in lactating dairy cows [17,21]. Aside from changing the body temperature, access to shade has also been shown to increase feed intake [22] and, consequently, milk production of dairy cows on pasture [17,21]. 

Although the beneficial effects of providing shade to heat-stressed cattle in hot climates have been well documented in both indoor and outdoor-kept dairy cows ([23,24]), little is known about how to shade access may influence the behavior and body fat mobilization of dairy cows throughout the dry period in temperate regions. Therefore, our study aimed to evaluate the effects of providing access to artificial shade on lying, rumination, feeding, and drinking behavior of prepartum dairy cows housed outdoor during a temperate summer. Additionally, the effects of shade access on energy analytes in the blood (prepartum NEFA and postpartum BHB) and clinical disease occurrence after calving were also evaluated. We hypothesized that prepartum cows with access to a shade would spend more time lying, ruminating, and feeding, and less time drinking in comparison to prepartum cows without shade access. We also predicted that energy analytes in the blood and disease occurrence would be reduced in dairy cows with prepartum shade provision.

## 2. Materials and Methods

This study was carried out between January and March 2019 during the summer season in southern Chile, at the Austral Agricultural Experimental Station of the Austral University of Chile, located in Valdivia (39°46′42′′ S, 73°13′38′′ W). Valdivia has a Temperate Oceanic Climate (Cfb: without a dry season and warm summer) according to Köppen-Geiger Classification [25]. The experimental farm managed a total of 160 Holstein-Friesian cows with an average of 305-d milk production of approximately 7000 kg (4.56% fat and 3.51% protein). 

### 2.1. Animals, Management and Experimental Design

The study included 24 multiparous Holstein-Friesian dairy cows that were selected from a group of 40 pregnant dry cows, based on their expected calving date and with no signs of clinical health problems (i.e., lameness, injuries). There was a difference of 28 days between cows with the earliest and latest calving dates. The animal’s parity number was [mean ± standard deviation (SD)] 3.3 ± 1.3, and the bodyweight (BW) and body condition score (BCS) at the start of the experiment was 620 ± 80 kg and 3.2 ± 0.4 on a 5-point scale, respectively. 

From the dry-off (60 days before their expected calving date) to approximately 30 days before the expected parturition date (far-off group), cows were housed together in paddocks with pasture (mixture of grasses and legumes) and managed daily in a rotational grazing method. Cows had free access to water bins and no access to shelter. The stocking rate in these paddocks was maintained at approximately 102 cow/ha. 

Approximately 30 ± 5 days before the expecting calving date, cows were allocated into six groups (4 cows per group) based on parity and BW, and each group was randomly assigned to one of two treatments: corrals without shade access (3 groups/*n* = 12 cows) or corrals with shade access (3 groups/*n* = 12 cows). All cows were moved at the same time from the far-off group to the experimental prepartum groups and were evaluated during the last 3 weeks of gestation. Data obtained during the first 4 days were excluded from the final analysis because that time was used to acclimatize the groups of cows.

**Figure 1:**
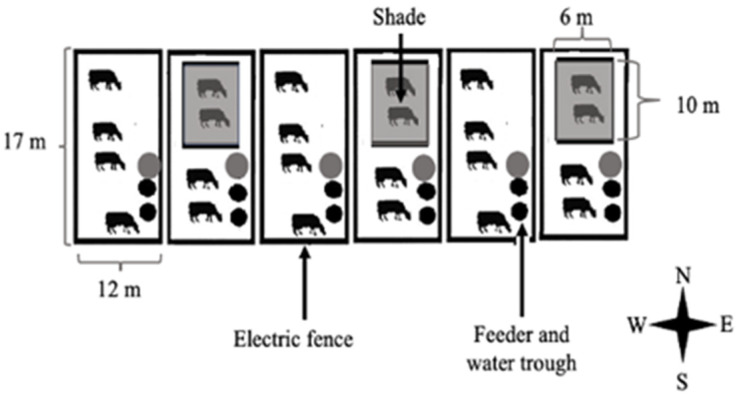
Illustration (not to scale) of the experimental design. Shade (grey rectangles) was oriented from the north toward the south. Feed (black circle) and water (grey circle) were located outside the shaded area.

The corrals in both treatments measured 17 m × 12 m (51 m^2^/cow), had a bare soil surface with no grass cover, and were delimited by an electric fence allowing visual and auditory contact but limiting tactile interaction between cows. A freestanding shade structure (10 m long, 6 m wide, and 2.5 m above the ground) was constructed in 3 out of the 6 corrals. The structure’s tops were covered with a shade cloth that blocked 80% of solar radiation, providing a shaded area of 15 m^2^/cow (measured between 10:00 h and 18:00 h). Shade structures were orientated in the same direction with the 6 m side facing north. Corrals with shade access were located interspersed among the corrals without shade access to prevent any overlap in shadow casting (Figure 1). All corrals were daily cleaned of manure in the morning during the study period.

Cows were fed twice a day at 08:30 and 18:00 h and the ration was provided in two feed bins placed outside the shaded area. The ration was formulated following the NRC, [25] guidelines and consisted of grass silage (40.5 ± 5.1% DM, 10.5 ± 1.7% CP, 54.8 ± 7.3% NDF and 10.9 ± 0.67 MJ/kg DM [mean ± SD]) and ~3 kg of commercial concentrates/d on an as-fed basis (87% DM, 22.5% CP, 12.0% NDF, and 11.56 MJ/kg DM [mean]) with anionic mineral mix (Mg 4.0%, Cl 33.0%, S 2.6%, Ca 0.7%, K 0.3%, 2 mg Na kg^−1^, 1050 mg Cu kg^−1^, 2100 mg Mn kg^−1^, 3500 mg Zn kg^−1^, 140 mg I kg^−1^, 13 mg Co kg^−1^, 10 mg Se kg^−1^) at a rate of 0.20 kg cow per day. Water was provided ad libitum in a water trough (one in each corral, 600 L).

After calving, cows remained in the experimental corrals for 6 h with their newborn calf. Then the calf was moved to the calf barn and the dam to the lactation group. The newly calved cow was not replaced by another cow to prevent changes in the social structure of the group. The experimental cows in the postpartum lactation group continued to be monitored until 21 days in milk (DIM). These cows were fed under a daily rotational grazing system conformed by perennial ryegrass and white clover (21.1% DM, 19.3% CP, 47.1% NDF, and 11.7 MJ/kg DM [mean]), providing 6 kg of commercial concentrates/d on an as-fed basis (86 to 88% DM, 11.5% CP, 32.3% NDF, and 13.19 MJ/kg DM [mean]) during each milking. A mineral mix (Ca 14.0%, P 10.0%, Mg 6.0%, Na 4.0%, S 0.2%, 5000 mg Zn kg^−1^, 1500 mg Cu kg^−1^, 200 mg I kg^−1^, 20 mg Co kg^−1^, 14 mg Se kg^−1^) was offered with the concentrate at a rate of 0.25 kg cow per day. All the animals were milked twice a day (at 06:00 and 15:00 h).

### 2.2. Data Collection

#### 2.2.1. Environmental Measurements

Climatic variables including air temperature (°C), relative humidity (%), wind speed (km/h), solar radiation (W/m^2^), and precipitations (mm) were recorded continuously using a weather station (A720, ADCON Telemetry GMBH) located 1 km from the research location. Data were recorded every 60 min over a 24-h period during the prepartum experimental period. These data were used to calculate THI and Heat Load Index (HLI) for each prepartum day, according to Ravagnolo and Misztal, [26] and Gaughan et al. [27], respectively. For the calculation of black globe temperature, we used the formula reported by Hahn et al. [28]. We then categorized each hour based on THI levels (<68 = no heat stress, and ≥68 = heat stress) [29,30], and HLI levels (<70 = thermoneutral conditions, and ≥70 = warm/hot conditions) [27].

#### 2.2.2. Behavioral Measurements

To determine the use of shade as well as the behavior under the shade (lying/standing), two infrared video cameras (Ezviz CS-CV310-A0-1B2WFR, Ezviz, City of Industry, CA, USA) were mounted on two corners of each corral at a height of 2.2 m, and both directed down the center of the shade structure. Video recordings were analyzed over the prepartum period using scan sampling at 3-min intervals during the hottest part of the day (time with the highest solar radiation) between 11:00 and 17:59 h. Prior to the video recording, each cow was marked with a unique number on both sides using hair dye to facilitate individual recognition. A cow was considered to be using the shade when at least 2 hooves were in the shade cast by the structure [18]. Lying underneath the shade was defined as a cow having its flank in contact with shaded ground, with no weight supported by any of the legs. Standing underneath the shade was defined as a cow in an upright posture without the forward motion in the shaded area. One trained observer transcribed the behavioral data from the videos, and the intra-observer percentage agreement was above 98% for all behaviors (shade use and lying/standing under shade). These data were used to calculate a percentage of daily use of the shade. 

Daily lying time, number of lying bouts per day (i.e., frequency of transitions from lying to standing positions), and duration of lying bouts per day (i.e., calculated as the ratio of minutes lying to the number of lying bouts per day) were measured using electronic data loggers (HOBO Pendant G Acceleration Data Logger, Onset Computer Corporation, Bourne, MA, USA, validated by Ledgerwood et al. [31]. The devices were configured to record at 1-min intervals and then each data logger was attached to the lateral side of one of the hind legs of the cow, along with a self-adhesive Coban™ self-adherent wrap, and remained in position up to three weeks after calving. Every 10 days, the data loggers were changed and the information from those days of change was not considered for the analysis. The data were downloaded weekly, isolated standing or lying events were removed from the base following the recommendation of Ledgerwood et al. [31].

Rumination time was recorded using an individual rumination tag on a neck collar. This automatic system (Hr-Tag, SCR Engineers Ltd., Netanya, Israel) records rumination time continuously over a 24-h period as validated for dairy cows by Schirmann et al. [32]. Data were stored in the memory of the logger and summarized as min/2-h intervals. The tag information was transferred to and stored in the control unit through radio frequency. This information was downloaded onto the database weekly. If one 2-h interval was not recorded by the system, the entire day was discarded.

Feeding and drinking behavior was recorded using one infrared trail camera (Q-See QT5682-411, Anaheim, CA, USA) situated 2 m above each feeder and water bin and fixed to stands. Both behaviors were evaluated on four days of each prepartum week (12 days/cow), using scan sampling every 3 min for each cow. Before the start of the experiment, we perform a preliminary study with other animals to measure the time that cows spend eating the morning and afternoon ration in order to define our observational period for assessing this behavior. Cows consumed the total delivered feed within 2.5 h in the morning and the afternoon. For this reason, to assess feeding time, video recordings were analyzed for 3.5 h after morning feeding (from 08:30 to 12:00 h) and for 3.5 h after afternoon feeding (from 18:00 to 21:30 h). A cow was considered as feeding when it was standing and having the head above/or in the feed bin, and silage was visible in her mouth as described by Schütz et al. [33]. To assess drinking time, video recordings were analyzed between 11:00 and 18:00 h. A cow was considered as drinking when she was standing and facing the water throw and swallowing water as described by Huzzey et al. [34]. Videos were analyzed by a one trained observer (intra-observer reliability was 96% for feeding behavior and 97% for drinking behavior). 

#### 2.2.3. Blood Sampling and Health Records

Blood samples from all cows were taken weekly during the same day from the time of enrollment until 3 weeks after parturition. During the prepartum period, between days −21 to −15 to represent week −3, days −14 to −8 to represent week −2, and days −7 to −2 to represent week −1 relative to calving. Postpartum blood samples were collected between days 2 to 7 to represent week 1, days 8 to 14 to represent week 2, and days 15 to 21 ± 1 to represent week 3. Samples were collected at approximately 09:00 h during the prepartum period and after morning milking during the postpartum period. Blood was collected from the coccygeal vein through vacutainer^®^ system into 5-mL EDTA sterile tubes and then transported to the laboratory within 5 h of collection. Serum was separated immediately upon arrival at the laboratory and stored at −20 °C until analysis. NEFA concentration (Randox, Crumlin, UK) in weeks −3, −2, and −1 before calving and BHB concentrations (Ranbut, Randox, Crumlin, United Kingdom) in weeks 1, 2, and 3 after calving were measured by enzymatic analysis using an auto-analyzer (Metrolab 169 2300, Wiener Lab, Rosario, Argentina). The intra-assay coefficient of variation for the BHB and NEFA assay were 3.8% and 2.8%, respectively. 

Clinical disease diagnosis was performed on each cow by a trained veterinarian on days 0, 3, 6, 9, 12, and 21 after calving, and after morning milking. Clinical examination included direct observation of the animal, measurement of the rectal temperature, ruminal auscultation, and evaluation of the vaginal discharge. The retained placenta was defined as failure to expel the placenta within 24 h after parturition. Clinical hypocalcemia was defined as any recumbent cow within 72 h after parturition exhibiting anorexia, nervous symptoms, staggering, varying degrees of unconsciousness, and good response to intravenously administered calcium. Puerperal metritis was characterized by a fetid watery red-brown vaginal discharge and rectal temperature ≥ 39.5 °C. Clinical mastitis was detected by the milker using inspection of the foremilk and palpation of the udder at milking and was characterized by the presence of abnormal milk or by signs of inflammation in 1 or more quarters. The presence of any other clinical health disorders such as digestive or respiratory problems was also recorded. Additionally, the gait score of the cows was assessed by the trained veterinarian when they exited the milking parlor using a 5-point numerical rating system (NRS), where 1 = sound and 5 = severely lame [35]. Lameness was categorized as clinical lameness (prevalence of cows scored as NRS = 3) and severe lameness (prevalence of cows scored as NRS = 4 or 5).

### 2.3. Statistical Analysis

One cow in the group without shade access was excluded from the experiment during the acclimatization period due to the display of frequent agonistic behavior (butting, pushing, fighting, and threatening) against other cows and its data was excluded from the analyses. 

Statistical analysis was performed using R language [36] with cow as the experimental unit. Environmental (temperature, relative humidity, wind speed, solar radiation, THI, and HLI) and behavioral measures (shade use, behavior under the shade, and rumination, feeding and drinking behavior) were summarized for each day and then summarized by observational periods based on days relative to parturition (week −3: days −21 to −15; week −2: days −14 to −8; week −1: days −7 to −2). Blood analytes values were stratified according to the prepartum (NEFA) and post-partum week (BHB) in which they were evaluated.

Mixed models, using the lme4 package [37] and lmerTest [38] was used to determine differences in the use of shade between the weeks prior to calving. The model included the fixed effects of the period relative to calving and the random effect of cow nested within the group.

To determine the effect of climate conditions on time spent under the shade, data of daily weather conditions were combined with shade use data for each cow according to the calendar date. Data were analyzed using mixed models (lme4 package and lmerTest), considering as fixed effects the period relative to calving and the daily weather variables (solar radiation, wind speed, THI, HLI, and their interactions), and as the random effect, the cow nested within the group. Temperature and relative humidity were strongly related (r > 0.8) to THI and, therefore, excluded from the model. The calculated AIC of the overall model determined whether additional climate factors improved the model fit. 

To detect differences in behavior and blood metabolite concentrations between cows with and without shade access during the prepartum period, data were analyzed using mixed models (lme4 package and lmerTest). The models considered treatment group (with and without shade access), period relative to calving and their interactions as fixed effects, and cow nested within the group as a random effect. The association between treatments (with and without shade) and health outcomes (absence or presence of health events) were analyzed using 2 × 2 contingency tables and Fisher’s exact test statistic.

Residuals were examined in all models to verify normality and homogeneity of variances and to detect possible outliers and influential points. No observations were removed from the analyses. The significance level was defined as *p* < 0.05, *p* < 0.01, *p* < 0.001, and tendencies were considered *p* < 0.1.

## 3. Results

### 3.1. Environmental Conditions

**Figure 2:**
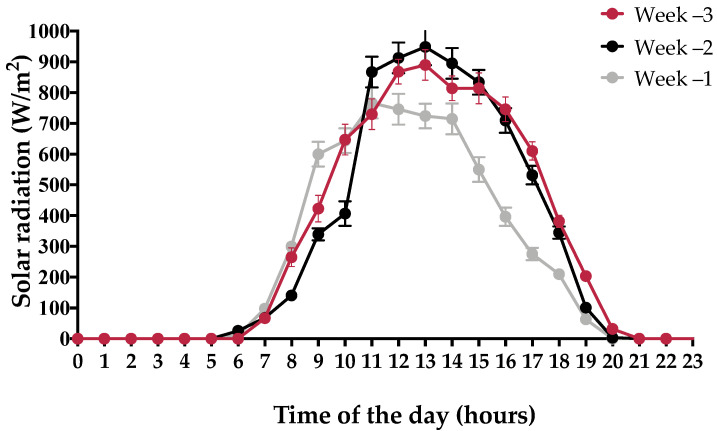
Mean (±SE) hourly solar radiation in different weeks prior to calving.

A summary of the weather conditions during the prepartum period is shown in Table 1. No precipitation was recorded on any of the experimental days. The percentage of hours with THI values ≥ 68 between 11:00 to 17:59 h was 86 ± 10%, 63 ± 8.6%, and 43 ± 11% during week −3, −2, and −1 relative to calving, respectively. During the same period of the day, the percentage of hours with HLI values >70 was 91 ± 7.1%, 72 ± 6.3%, and 50 ± 9.2% during week −3, −2, and −1 relative to calving, respectively. Hourly means of solar radiation during the study period are shown graphically in Figure 2.

### 3.2. Shade Use 

During 11:00 to 17:59 h, we found that cows spent approximately half of their time beneath the shade during week 3 (45.6 ± 2.7%; range: 10–96%) and 2 (46.0 ± 3.0%; range: 11–100%) prior to calving. However, shade use was lower on the week prior to calving than on previous weeks (19.8 ± 2.1%, range 0–67%; *p* < 0.05). On average, cows spent a low proportion of their time lying down beneath the shade during week 3 (10.6 ± 2.1%, range 0–25%), and 2 (8.9 ± 1.3, range 0–19%) before calving, and during the week prior to calving was even lower than on previous weeks (6.4 ± 1.2%, range 0–21%; *p* < 0.1). Most of the time, prepartum cows used the shade individually (30.1 ± 1.7%) or in pairs (36.7 ± 2.0%). Only 21.5 ± 2.3% and 11.7 ± 1.7% of the time we observed three or four cows using the shade simultaneously, respectively. 

**Figure 3:**
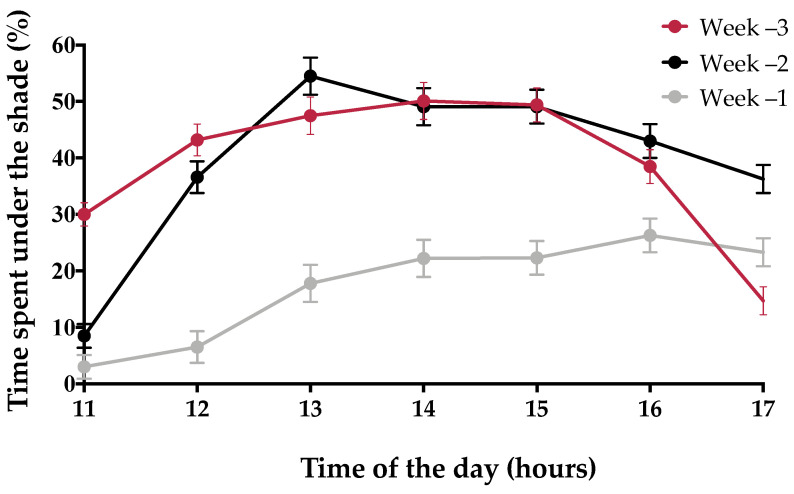
Mean (±SE) percentage of time prepartum cows spent beneath the shade in different weeks prior to calving (*n* = 12). The results are shown for 60-min intervals from 11:00 h to 17:59 h.

Prepartum cows used the shaded area at different hours of the day, reaching the highest values between 13:00 and 15:00 h during weeks 3 and 2 prior to calving (Figure 3). The use of the shade by cows was influenced by some environmental conditions during the entire prepartum period; the increase in HLI and solar radiation increased the use of shade (HLI Estimate: 0.7%; SE: 0.02; *p* < 0.01; solar radiation Estimate: 0.1%; SE: 0.02; *p* < 0.05), and the wind speed decreased the time that cows spent lying under the shade (Estimate: −6.4%; SE: 2.0%; *p* < 0.01). THI had no effect on the cow location and the posture under the shade (lying/standing).

### 3.3. Lying, Ruminating, Feeding, and Drinking Behavior

Treatments had no effect on total daily lying time, lying bout, and lying bouts duration pre-calving (*p* > 0.05; Table 2). Overall, we observed a week effect in which cows spent less time lying and had more lying bouts of shortening duration during the week prior to calving than in other weeks, regardless of the treatment (*p* < 0.01). There was a tendency for cows with shade access to have a greater mean daily rumination time during week 3 prior to calving compared to cows without shade access (*p* < 0.09; Table 2).

Between 11:00 to 17:59 h, cows with access to shade spent less time drinking than cows without shade access (Table 3). Although cows in both treatments consumed all the feed within the morning and evening observation periods, cows with access to shade spent more time feeding than cows without shade access only during the morning hours (Table 3).

### 3.4. NEFA, BHB, and Health Status

We found no treatment effect on NEFA or BHB concentrations pre-calving (*p* > 0.05; Table 4). The proportion of cows that were considered as clinically ill or healthy after calving was similar between treatments, where 33% (*n* = 4) of cows with shade access (*n* = 12) and 27% (*n* = 3) of cows without shade access (*n* = 11) were diagnosed with at least one clinical disease postpartum (Fisher´s exact test, *p* = 0.99). 

## 4. Discussion

Although the importance of shade access for cattle exposed to summer weather conditions in temperate climates has been the focus of several studies (reviewed by Van Laer et al., [39] and Webster et al., [40]), to our knowledge no studies have described the effectiveness of providing shade for prepartum dairy cows managed outdoor. We found that when prepartum cows had free access to an artificial shade during the warmest hours of the day, they spent approximately half of this time beneath the structure (~3.2 h; from 11:00 to 18:00 h), especially in the third and second week prior to calving. This result is in agreement with previous findings that mid-lactating cows on pasture used the shade approximately 3 h during the same period of the day and under similar weather conditions in New Zealand [18,20]. However, our results showed a decrease in shade use of approximately 20% (1.4 h) during the week prior to calving. This finding was most likely because during this week the percentage of hours under hot weather conditions was lower (THI 43% and HLI 50%) compared to the other weeks (week −3, THI 86% and HLI 72%; week −2, THI 63% and HLI 70%). When prepartum cows were beneath the shade between 11:00 and 18.00 h, they spent less than 10% of the time lying down. It is possible that the motivation for lying during the hottest period of the day decreases as an attempt to dissipate the heat gained from the environment and the fetus [41], since a standing position increases the available body surface for heat dissipation [17,42,43]. 

The use of shade and the behavior beneath the shade (i.e., standing/lying) were influenced by environmental conditions, specifically solar radiation and wind speed. Regardless of the week relative to calving, cows spent more time under the shade as the average solar radiation increased, showing that prepartum cows try to reduce body temperature by seeking shade, in the same way, it has been described for lactating cows [17,18,20]. Providing prepartum cows with shade is likely beneficial in terms of reducing heat load due to the protective effect of these structures on solar radiation (reviewed by Lees et al., [44]). We also observed that the wind speed reduced the time that cows remained lying under the shade. This finding disagrees with Tullo et al., [45] who observed longer lying times in housed lactating dairy cows as the wind speed increase under a shade roof from 1 m/s to 1.8 m/s. These authors suggested that air movement underneath the shade favors the dissipation of heat to the environment, generating a cooler environment and promoting lying behavior. It is possible that in our study the artificial shade did not create a cool environment under it, favoring the cows to remain standing position exposing their body to the wind. Unfortunately, we did not measure the floor temperature under the shade to test this hypothesis. A better understanding of the effects of environmental conditions on shade use and behavioral responses in prepartum dairy cows is needed. 

In our study, all cows used the shade but most of the time they were alone (30%) or in pairs (37%). One possible explanation might be that the shaded area was a limited resource for the group, leading to agonistic interactions [20,46]. However, the space granted in our experiment (15 m^2^/cow) was higher than the 3.5 to 5.6 m^2^/cow recommended for mature dairy cows [47]. Schütz et al., [48] observed a similar behavior, where lactating dairy cows rarely used simultaneously the shade in pasture paddocks, even at low densities (8.1 and 8.8 m^2^/cow). In our study, factors such as hierarchy or social isolation, which is important in cows close to calving [49], may have influenced this behavior and are necessary to consider in future research.

The daily lying time was similar for shaded and unshaded cows (10.7 and 11.2 h, respectively) over the prepartum weeks which agrees with previous studies carried out in lactating grazing dairy cows with and without shade access under temperate summer weather conditions [7,18,19]. Previous studies have found that temperatures below 21 °C overnight for 3 to 6 h allow the opportunity for the dairy cattle to dissipate the heat gained during the day [50]; thus, there is evidence that during the warm day weather conditions dairy cattle are motivated to lie down during the cooler night hours [7]. We did not investigate lying time during the day and night hours separately in this study, but we argue that both groups of cows were motivated to lying during night hours, independently of the shade access during the day. We also found that access to shade did not affect the daily lying bouts or bouts duration during the prepartum weeks. However, we observed that during the week previous to calving the daily lying bouts increased and lying bouts duration decreased significantly in both groups. These findings are in line with recent studies that found an increase in lying bouts and a reduction in the duration of lying bouts of healthy grazing dairy cows in the days close to calving [51,52]. These changes in lying patterns might be an indicator of discomfort and restlessness associated with parturition [34,52].

Shaded cows tended to have a higher rumination time compared to non-shaded cows (~59 min) during the prepartum period. Shorter rumination times have been described in housed lactating cows under warm (THI > 76; Soriani et al., [53]) and temperate (THI > 52; [54]) environment conditions. However, others have shown no relationship between rumination time and THI conditions [55]. Paudyal et al., [9] observed that rumination time in prepartum cows was shorter in the hot season (monthly average THI ≥ 76) than in the cool season (monthly average THI < 76; 432 vs. 487 min/d) under subtropical weather. Further research is encouraged to determine the effect of shade access on the rumination behavior of cows kept in outdoor conditions during the prepartum period in temperate regions.

Feeding time tended to be higher in shaded cows during morning feeding compared to unshaded cows, and the reason for this remains unclear. Moallen et al., [56] and Soriani et al., [53] observed reductions in the dry matter intake of lactating dairy cows in the next days when exposed to THI values > 76. In the present study, although the dry matter intake was not evaluated, shaded and unshaded cows ate the whole ration both in the morning and evening, affecting only the feeding time. In this sense, small but more frequent meals are described in lactating dairy cows exposed to heat stress [57], which could have reduced the feeding time in cows without access to shade. Also, we hypothesized that cows without access to shade generated frustration in the animals, as has been previously reviewed by Polsky and von Keyserlingk [58]. This could increase competition in the feeder and, therefore, a reduction in feeding time as reported by Crossley et al., [59]. However, this was not evaluated in the present study.

Drinking time during hot hours of the day (11:00–17:59) tended to be twofold higher in cows that had no access to shade when compared to shaded cows throughout the prepartum period. This result is in line with previous studies carried out under grazing conditions, where lactating cows without access to shade increased the time spent near a water trough [7,20]. The increase in the amount of water in unshaded cows could be related to increased water loss via skin and respiratory evaporation to dissipate the body heat [60], so shade access could be beneficial in reducing water demands. During the period around calving, the benefits of shade access may be greater because during this period cows are more thirsty because of exhaustion, stress, and colostrum production, leading to long drinking times such as the case of early lactation [61].

We did not observe any effect of access to shade on serum NEFA concentrations in cows during the weeks before calving. This result might be explained by the relationship between NEFA concentration and food consumption [62], and both shaded and non-shaded cows consumed the whole ration of food, varying only the time spent eating the meal. It is also possible that due to the few heat stress events that the cows faced within this experiment, these animals had fewer energy costs associated with thermoregulation [63].

Similarly, we found no differences in BHB concentrations between cows with access and cows without access to shade during the prepartum period. Taken together, these results suggest that access to shade under moderate warm weather conditions did not affect fat mobilization in prepartum cows. These findings could have also influenced the health results because in both groups NEFA and BHB concentrations were similar or lower than the limits proposed by Ospina et al., [14] as a risk factor for the development of postpartum diseases (i.e., clinical ketosis, abomasal displacement, placental retention, and metritis).

## 5. Conclusions

The results from this study showed that access to an artificial shelter during the last 3 weeks of the dry period under temperate summer conditions facilitated the daily rumination time, increased the feeding time after morning food delivery, and decreased drinking time, but it did not have a significant effect on NEFA or BHB blood concentrations or health status after calving. These results provide evidence that when prepartum cows are kept outdoors during the autumn calving season, cows should be given access to shade to reduce any negative effects of the warm weather on their behavior.

## Figures and Tables

**Table 1:** Weekly summary (mean, Standard Error (SE), range) of summer weather variables recorded over 24 h and from 11:00 to 17:59 h during three weeks before calving.

Week Relative to Calving	Weather Variable	24 h			11:00–17:59 h		
		Mean	SE	Range	Mean	SE	Range
−3	Temperature (°C)	16	0.8	9–24	22	0.6	20–24
	Relative Humidity (%)	70	2.1	37–90	49	1.9	37–60
	Wind Speed (m/s)	2	0.2	0–4	3	0.1	2–3
	Solar Radiation (W/m^2^)	256	11.4	0–802	776	2.1	737–802
	THI ^1^	61	0.5	47–70	67	0.4	65–70
	HLI ^2^	57	0.4	42–65	72	0.3	69–76
−2	Temperature (°C)	17	1.0	8–35	26	0.7	20–35
	Relative Humidity (%)	68	1.8	23–85	39	1.7	23–53
	Wind Speed (m/s)	2	0.3	0–4	3	0.2	3–4
	Solar Radiation (W/m^2^)	300	10.3	0–792	769	1.8	754–792
	THI ^1^	63	0.6	51–75	70	0.6	65–75
	HLI ^2^	58	0.4	47–72	75	0.5	67–79
−1	Temperature (°C)	15	0.8	8–23	22	0.4	21–23
	Relative Humidity (%)	70	1.8	29–76	40	1.7	29–63
	Wind Speed (m/s)	2	0.3	0–4	3	0.2	3–4
	Solar Radiation (W/m^2^)	285	11.0	0–778	734	1.7	742–778
	THI ^1^	59	0.2	45–66	64	0.1	64–66
	HLI ^2^	56	0.3	43–62	67	0.3	67–71

^1^ THI—temperature–humidity index. ^2^ HLI—heat-load index.

**Table 2:** Daily lying time, number of lying bouts, lying bouts duration, and rumination time (0000–2359 h) were observed in cows with access to shade (*n* = 12) and without shade (*n* = 11) in different weeks prior to calving (LSM ± SE). *p*-values are presented by prepartum week, between treatments.

Behavior	Shade	without Shade	*p*-Value
Lying time (min/d)			
Week −3	644 ± 21	681 ± 19	0.50
Week −2	671 ± 24	698 ± 21	0.55
Week −1	642 ± 16	626 ± 20	0.62
Lying bouts (*n*/d)			
Week −3	14 ± 0.8	14 ± 0.6	0.59
Week −2	15 ± 0.6	16 ± 0.8	0.34
Week −1	16 ± 0.9	17 ± 1.3	0.54
Bout duration (min/bout)			
Week −3	51 ± 3.8	50 ± 2.5	0.82
Week −2	49 ± 3.1	48 ± 3.0	0.62
Week −1	44 ± 3.9	41 ± 3.1	0.50
Rumination time (min/d)			
Week −3	532 ± 23	466 ± 32	0.09
Week −2	529 ± 20	472 ± 32	0.21
Week −1	513 ± 16	461 ± 29	0.19

**Table 3:** Daily drinking time (1100–1759), morning feeding time (0830–1159 h), and evening feeding time (1800–2030 h) were observed in cows with access to shade (*n* = 12) and without shade (*n* = 11) in different weeks prior to calving (LSM ± SE). *p*-values are presented by prepartum week, between treatments.

Behavior	Shade	without Shade	*p*-Value
Drinking (min/7 h)			
Week −3	6.8 ± 0.8	16.4 ± 3.7	0.04
Week −2	8.5 ± 1.3	14.7 ± 2.7	0.09
Week −1	8.0 ± 1.1	15.5 ± 4.0	0.08
Morning feeding (min/3.5 h)			
Week −3	72.6 ± 4.1	59.4 ± 4.6	0.10
Week −2	81.3 ± 5.5	61.1 ± 2.7	0.02
Week −1	86.3 ± 6.1	69.7 ± 3.3	0.05
Evening feeding (min/3.5 h)			
Week −3	56.1 ± 3.7	54.8 ± 1.4	0.76
Week −2	54.9 ± 5.9	61.5 ± 1.9	0.31
Week −1	59.9 ± 3.6	57.2 ± 2.5	0.62

**Table 4:** Serum NEFA and BHB concentrations were observed in cows with access to shade (*n* = 12) and without shade (*n* = 11) in different weeks relative to calving (LSM ± SE). *p*-values are presented by prepartum and postpartum week, between treatments.

Metabolite	Shade	Without Shade	*p*-Value
NEFA (mmol/L)			
Week −3	0.26 ± 0.04	0.32 ± 0.05	0.37
Week −2	0.25 ± 0.05	0.30 ± 0.06	0.42
Week −1	0.35 ± 0.05	0.34 ± 0.06	0.86
BHB (mmol/L)			
Week 1	0.74 ± 0.08	0.93 ± 0.13	0.20
Week 2	0.78 ± 0.07	1.10 ± 0.21	0.25
Week 3	0.70 ± 0.10	0.86 ± 0.13	0.58

## Data Availability

The data can be found at: https://doi.org/10.6084/m9.figshare.16767277.v1.
